# Transcriptional Shifts Highlight the Role of Nutrients in Harmful Brown Tide Dynamics

**DOI:** 10.3389/fmicb.2019.00136

**Published:** 2019-02-12

**Authors:** Louie L. Wurch, Harriet Alexander, Kyle R. Frischkorn, Sheean T. Haley, Christopher J. Gobler, Sonya T. Dyhrman

**Affiliations:** ^1^Department of Biology, James Madison University, Harrisonburg, VA, United States; ^2^Department of Biology, Woods Hole Oceanographic Institution, Woods Hole, MA, United States; ^3^Department of Earth and Environmental Sciences, Lamont-Doherty Earth Observatory, Columbia University, Palisades, NY, United States; ^4^School of Marine and Atmospheric Sciences, Stony Brook University, Stony Brook, NY, United States

**Keywords:** harmful algal bloom, *Aureococcus anophagefferens*, brown tide, nutrient physiology, metatranscriptomics

## Abstract

Harmful algal blooms (HABs) threaten ecosystems and human health worldwide. Controlling nitrogen inputs to coastal waters is a common HAB management strategy, as nutrient concentrations often suggest coastal blooms are nitrogen-limited. However, defining best nutrient management practices is a long-standing challenge: in part, because of difficulties in directly tracking the nutritional physiology of harmful species in mixed communities. Using metatranscriptome sequencing and incubation experiments, we addressed this challenge by assaying the *in situ* physiological ecology of the ecosystem destructive alga, *Aureococcus anophagefferens*. Here we show that gene markers of phosphorus deficiency were expressed *in situ*, and modulated by the enrichment of phosphorus, which was consistent with the observed growth rate responses. These data demonstrate the importance of phosphorus in controlling brown-tide dynamics, suggesting that phosphorus, in addition to nitrogen, should be evaluated in the management and mitigation of these blooms. Given that nutrient concentrations alone were suggestive of a nitrogen-limited ecosystem, this study underscores the value of directly assaying harmful algae *in situ* for the development of management strategies.

## Introduction

Harmful algal blooms (HABs) are a global concern in both freshwater and coastal ecosystems, and the frequency and intensity of HABs have been increasing worldwide (Anderson et al., 2002; Heisler et al., 2008; Fu F.X. et al., 2012). Such proliferations of harmful algae can threaten human health, degrade ecosystems, and cost hundreds of millions of dollars in mitigation, management, and lost revenue (Anderson et al., 2002; Gobler et al., 2005; Anderson et al., 2008; Anderson et al., 2012; Gobler and Sunda, 2012). Nutrients such as nitrogen (N) and phosphorus (P) play an important role in HAB dynamics, with the current paradigm suggesting that N is the primary driver of marine coastal HABs while P has a larger role in controlling freshwater HABs (Ryther and Dunstan, 1971; Michalak et al., 2013). This paradigm influences management and mitigation strategies, which typically focus on one nutrient or the other (Ryther and Dunstan, 1971; Conley et al., 2009; Paerl et al., 2016). Identifying controls on HABs is challenging, as there are few methods that can specifically assess the dynamic physiological status of a single harmful algal species in a mixed plankton community. Further, direct measurements of nutrients are often not related to cell dynamics because bioavailability and flux to a particular species cannot be quantitatively constrained (Dyhrman, 2008). These challenges have led to uncertainty regarding the role of nutrients in driving HABs (Heisler et al., 2008; Davidson et al., 2014), so a comprehensive characterization of the role of nutrients in HABs is vital for improved management. High-throughput transcriptome sequencing of plankton populations in an aquatic ecosystem setting (metatranscriptomics) offers one solution to this challenge, as it enables the high-resolution characterization of an organism’s response to its environment *in situ* within a mixed community. Metatranscriptome sequencing is increasingly being applied to studies of HABs, providing critical information about microbial community structure changes, nutrient utilization strategies, and diel cycling patterns, among other factors (Zhuang et al., 2015; Gong et al., 2017; Ji et al., 2018).

Here we used metatranscriptome sequencing combined with *in situ* nutrient manipulations to directly examine nutrient controls on a particularly devastating HAB caused by the alga *Aureococcus anophagefferens*. This alga is responsible for ecosystem-disruptive blooms that result in shellfish mortality, destruction of habitat, alterations of food webs, and the loss of millions of dollars to local economies annually in collapsed fisheries (Gobler et al., 2005; Gobler and Sunda, 2012; Zhang et al., 2012). Cells of *A. anophagefferens* reach unusually high densities (>10^9^ L^-1^), discoloring water and resulting in so called “brown tides” that have recurred annually on the US East Coast since the 1980s (Anderson et al., 2008; Boneillo and Mulholland, 2013) and also occur in South Africa (Gobler et al., 2005) and China (Zhang et al., 2012). Although the term “brown tide” is general and can refer to blooms of other pelagophytes, here we specifically use the term “brown tide” to mean a bloom of *A. anophagefferens*.

Like many other marine HABs, *A. anophagefferens* blooms in shallow, anthropogenically-influenced estuaries when levels of light are low and organic carbon and N inventories are elevated (Sunda et al., 2006; Anderson et al., 2008). More than two decades of research have emphasized the importance of N to the occurrence and ecology of brown tides, with blooms occurring in estuaries where levels of dissolved organic N (DON) are high and dissolved inorganic nitrogen (DIN) are low (Mulholland et al., 2002; Gobler and Sunda, 2012). However, it has become clear that there are additional controls on bloom formation and termination (Gastrich et al., 2004; Gobler and Sunda, 2012), as nutrient concentrations, and/or nutrient ratios, have not always accurately predicted cell densities or bloom dynamics for this or other harmful species (Dyhrman, 2008).

*Aureococcus anophagefferens* was the first eukaryotic HAB species to have its full genome sequenced and annotated (Gobler et al., 2011), yielding important insights regarding the manner in which the species’ unique gene complement may allow it to thrive. The sequenced genome has facilitated gene expression studies of axenic cultures of *A. anophagefferens*, which has provided key information regarding this destructive alga’s physiological capabilities. For example, gene expression studies have highlighted the ability of *A. anophagefferens* to switch to organic nutrient sources when inorganic nutrients are limiting, its increased capacity for organic carbon utilization under low light, and the restructuring of cellular membranes to reduce P demands during P deficiency (Berg et al., 2008; Wurch et al., 2011b; Frischkorn et al., 2014). Still, effective prediction and management of brown tides has remained an elusive goal (Gobler and Sunda, 2012). To better understand possible constraints on bloom intensity and termination, global gene expression patterns were analyzed from environmental samples obtained during a brown tide that occurred in Quantuck Bay (NY) during 2011 and signals were contextualized with nutrient amendment experiments.

## Materials and Methods

### Experimental Design and Field Sampling

**FIGURE 1:**
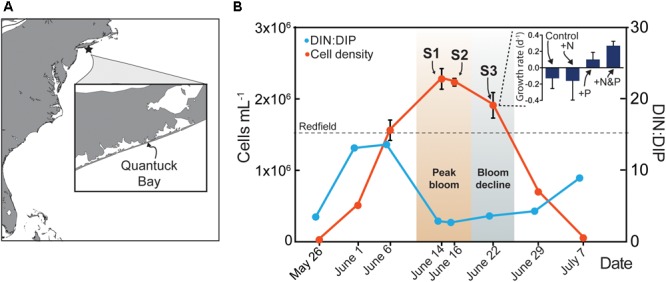
Map of study site in Quantuck Bay, NY where a severe brown tide occurred in 2011 **(A)** *A. anophagefferens* cell densities (red) over the course of the bloom **(B)** The surface water DIN:DIP ratio is indicated (blue) with the Redfield ratio (dashed line) indicated at 16. Arrows represent points in the bloom where metatranscriptome analyses were performed. Samples (S) S1 and S2 represent peak bloom conditions whereas S3 represents bloom decline. On June 22nd, a nutrient amendment (N, nitrogen; P, phosphorus) experiment was conducted and samples were taken after 24 h for metatranscriptome analysis. The growth rates of *A. anophagefferens* are shown in the embedded graph for this nutrient amendment experiment.

Samples were collected from a naturally occurring brown tide bloom that occurred in Quantuck Bay (Latitude = 40.806395 N; Longitude = 72.621002 W) from late May to early July in 2011 spanning the initiation, peak, and decline in *A. anophagefferens* cell numbers (Figure 1). Samples were collected at approximately the same time every day. A YSI© 556 Sonde was used to measure physical parameters such as temperature, salinity, and dissolved oxygen (Table 1). To obtain cell concentrations of *A. anophagefferens*, whole water was preserved with filter-sterilized 10% glutaraldehyde solution (1% final v/v), stored at 4° C, and subsequently analyzed using an enzyme-linked immunosorbent assay (ELISA) with a monoclonal antibody via an immunofluorescent flow cytometric technique (Stauffer et al., 2008). Planktonic chlorophyll *a* was measured fluorometrically (Welschmeyer, 1994) on 0.2 and 5 μm filters. Given its small size, *A. anophagefferens* only contributes to the <5 μm size fraction of chlorophyll *a* and its relative abundance was estimated among total phytoplankton using previously published cellular chlorophyll *a* quotas (Gobler et al., 2004; Table 1). Samples were collected for nutrient analysis by filtering seawater with acid-cleaned, polypropylene capsule filters (0.2 μm). Nitrate, nitrite, ammonium, and phosphate were measured in duplicate by standard spectrophotometric techniques (Jones, 1984; Parsons et al., 1984). Additionally, total dissolved N and P (TDN, TDP) were measured in duplicate by persulfate oxidation techniques (Valderrama, 1981). To calculate dissolved organic nitrogen (DON) and phosphorus (DOP), concentrations of nitrate, nitrite and ammonium or orthophosphate were subtracted from concentrations of TDN and TDP, respectively. Full recoveries (mean ± 1 S.D.) were obtained of samples spiked with SPEX Certi-PrepINC standard reference material at environmentally relevant concentrations of nitrate, nitrite and ammonium, phosphate, TDN, and TDP. Nutrient concentrations were compared to cell numbers using simple linear regression analysis.

**Table 1:** Compiled brown tide field sampling data for Quantuck Bay in 2011.

Date	*Aureococcus* (cells mL^-1^)	T (C°)	Salinity (psu)	NO_3_	PO_4_	NH_4_	SiO_4_	TN^∗^	TP^∗^	DON^∗^	DOP^∗^	DIN:DIP^∗^	DON:DOP^∗^	TN:TP^∗^
5–26	28666 ± 2138	17.1	28.7	0.24 ± 0.09	0.26 ± 0.05	0.66 ± 0.14	9.97 ± 0.47	41.09 ± 3.84	1.37 ± 0.30	40.2	1.11	3.43	36.22	29.97
6–1	510218 ± 44955	19.2	28.4	0.28 ± 0.02	0.14 ± 0.03	1.53 ± 0.65	28.02 ± 0.14	52.89 ± 0.53	1.23 ± 0.10	51.08	1.09	13.09	46.93	43.1
6–6	1561687 ± 143552	21.2	25.8	3.18 ± 0.34	0.26 ± 0.06	0.37 ± 0.18	53.10 ± 5.82	60.10 ± 8.20	1.21 ± 0.11	56.55	0.95	13.57	59.66	49.7
6–14	2280557 ± 144323	20.3	28.1	0.72 ± 0.12	0.38 ± 0.08	0.37 ± 0.06	58.13 ± 1.94	46.14 ± 0.63	1.22 ± 0.30	45.05	0.83	2.84	54.11	37.97
6–16	2234777 ± 53377	21.4	28.3	0.46 ± 0.02	0.31 ± 0.08	0.37 ± 0.30	70.43 ± 1.75	43.09 ± 3.13	1.30 ± 0.18	42.26	0.99	2.66	42.79	33.17
6–22	1910487 ± 179057	24.1	27.5	0.83 ± 0.08	0.33 ± 0.08	0.34 ± 0.06	88.95 ± 1.72	57.37 ± 6.90	1.48 ± 0.30	56.2	1.15	3.56	48.83	38.79
6–29	698864 ± 16712	25.5	27.3	0.89 ± 0.02	0.37 ± 0.10	0.69 ± 0.41	93.89 ± 2.35	51.59 ± 0.87	1.61 ± 0.28	50.01	1.23	4.24	40.58	32.14
7–7	54402 ± 505	26.1	28	0.48 ± 0.10	0.24 ± 0.06	1.67 ± 0.06	110.74 ± 4.45	53.21 ± 1.07	1.46 ± 0.04	51.06	1.22	8.89	41.89	36.42

*Nutrients are in μM. Numbers after ± indicate standard deviation. ^∗^DIN, dissolved inorganic nitrogen; DIP, dissolved inorganic phosphorus; DON, dissolved organic nitrogen; DOP, dissolved organic phosphorus; TN, total nitrogen; TP, total phosphorus.*

On June 22nd, during bloom decline, a nutrient amendment experiment was performed by filling 2 L bottles with natural seawater from the bloom and amending with the following nutrients in triplicate: 25 μM ammonium only (+N), 4 μM phosphate only (+P), and 25 μM ammonium and 4 μM phosphate (+N&P). Three additional bottles were filled and no nutrients were added (control). These bottles were placed in a floating chamber at 0.5 m in eastern Shinnecock Bay at the Stony Brook – Southampton Marine Science Center under one layer of neutral density screening, providing light and temperature levels that matched conditions within Quantuck Bay. The bottles were sampled for chlorophyll *a, A. anophagefferens* cell densities, and total RNA at T = 0 and T = 24 h following methods described above and below.

In addition, a culture experiment was performed using *A. anophagefferens* strains CCMP1984 and CCMP1850 obtained from the National Center for Marine Algae and Microbiota (NCMA). Triplicate axenic cultures were grown in batch at 18°C on a 14:10 h light: dark cycle (∼140 μmol quanta m^-2^ s^-1^) in L1 media with no Si (Guillard and Hargraves, 1993), prepared using 0.2 μm filtered Vineyard Sound seawater. Vitamins (thiamine, biotin and B12) were sterile filtered and added to the media after autoclaving. To monitor growth, fluorescence was tracked and cells were counted on a hemacytometer. Cells were harvested for sequencing during mid-log phase of growth as described below for field samples. The culture experiment for strain CCMP 1984 was used to provide a nutrient replete culture control for transcriptome comparisons, while CCMP 1850 was used to evaluate the potential influence of strain heterogeneity on read mapping.

### RNA Processing

Environmental RNA samples were collected from each bottle in the nutrient amendment experiment and from three time points in the bloom representing two samples at peak cell density (S1 and S2) and one sample as the bloom declined (S3) (Figure 1). Approximately 25 ml of natural seawater was obtained from Quantuck Bay for the environmental samples, or from the bottles for the nutrient amendment experiment, pre-filtered through 5 μm polycarbonate (PC) filters using low-pressure vacuum and cells were collected onto 0.2 μm polycarbonate filters (*A. anophagefferens* is a 2 μm cell). Samples were collected from the laboratory experiment in an identical manner except the 5 μm pre-filtration step was not used. In all cases, samples were flash frozen in liquid nitrogen within minutes of filtration and subsequently transferred to -80°C. CTAB buffer (Teknova), amended by 1% mass/volume with polyvinylpyrrolidone, was added to each sample before RNA extraction and RNA was isolated using the UltraClean^®^ Plant RNA Isolation Kit (MO BIO Laboratories), with modified manufacturer’s instructions for extraction from CTAB. To remove potential genomic DNA contamination, the RNA was treated with TURBO DNase according to manufacturer’s instructions for rigorous DNA removal (Ambion). Finally, the RNA was quantified spectrophotometrically for yield and purity. After isolation, RNA was pooled from triplicate filters for each sample. Approximately 4 μg of RNA was enriched using a poly A-selection, and sequenced using Illumina HiSeq 2000 at the Columbia Genome Center (New York, NY, United States), at a target depth of 50 million 100 bp single-end reads. A replicate sample from June 22 from a different set of triplicate filters was also sequenced at a target depth of 100 million reads.

### Metatranscriptome Analysis

Raw sequence data quality was visualized with FastQC and trimmed with Trimmomatic v.0.27 as previously described (Frischkorn et al., 2014; Alexander et al., 2015). Reads were mapped to the *A. anophagefferens* CCMP1984 genome^1^ using Tophat v. 2.0.4 with default parameters (Trapnell et al., 2009). Reads were similarly mapped to other genomes from potentially co-occurring organisms including: *Thalassiosira pseudonana, Phaeodactylum tricornutum, Ostreococcus tauri*, and *Ostreococcus lucimarinus* (Supplementary Table S1). Only reads that exclusively mapped to *A. anophagefferens* were included in this analysis. All project sequence reads are available at the National Center for Biotechnology Information (NCBI) under accession number SRP072764 and bioproject number PRJNA315054.

To evaluate the potential influence of strain heterogeneity on read mapping, reads were aligned from June 14 (S1) against assembled transcriptomes from two strains of *A. anophagefferens*, the genome isolate (CCMP1984) and an additional toxic isolate (CCMP1850) from the same region. The CCMP1984 RNA was sequenced as described above for field isolates, and CCMP1850 RNA was sequenced as described elsewhere (Frischkorn et al., 2014). The raw reads were trimmed from both strains using Trimmomatic (Lohse et al., 2012) and then normalized by k-mer coverage (max coverage 30) and assembled de novo using the Trinity software suite (Grabherr et al., 2011). Following assembly, the contigs were clustered at 90% identity using CD-Hit (Fu L. et al., 2012) after the procedures optimized by Frischkorn et al. (2014). The reads were then aligned from the June 14 field sample (S1) against the clustered and unclustered contigs from strain CCMP1984 and strain CCMP1850 with Bowtie2 using default settings (0 mismatches allowed).

To assign significance in differential expression, Analysis of Sequence Counts (ASC) was used (Wu et al., 2010), with a fold change greater than or equal to 2 and a posterior probability (post-*p*) > 0.95 as has been used in studies of similar design (Dyhrman et al., 2012; Thomas et al., 2012; Konotchick et al., 2013; Frischkorn et al., 2014; Alexander et al., 2015; Haley et al., 2017; Frischkorn et al., 2018). ASC is an empirical Bayes method that estimates the prior distribution by modeling biological variability using the data itself, rather than imposing a negative binomial distribution. ASC has been shown to perform similarly to, though more conservatively than, other differential expression analyses implemented on data sets with and without replicates (Wu et al., 2010). To assess reproducibility in our data, RNA was extracted from two independent samples from June 22. The RNA pool from the first sample was sequenced at a target depth of 50 million reads (S3a). The RNA pool from the second sample was sequenced at a target depth of 100 million reads (S3). Consequently, S3a and S3 represent replicated field samples sequenced at different depths. Changes in transcript abundance between samples were examined using ASC (fold change ≥ 2; post-*p* > 0.95). Only four genes had significant changes in abundance between S3a and S3 (biological replicates). For comparison, over 2000 genes had significant changes in abundance between S1 (June 14) and S3 (June 22). These data emphasize that the sequencing and analysis are reproducible among independent replicates.

Using a Kyoto Encyclopedia of Genes and Genomes (KEGG) framework, global expression patterns were generated as per Alexander et al. (2015). In brief, KEGG annotations were obtained from the Joint Genome Institute (JGI)^2^. KEGG annotations were associated into higher level functional categorizations based on KEGG-defined BRITE hierarchy ko00002 (Kanehisa et al., 2008). Many genes are associated with multiple different metabolic pathways, thus if a gene was present in more than one pathway it was counted in all pathways. Reads mapping to a KEGG id were normalized to total library size and then normalized to the summed reads with KEGG annotation.

## Results

The environmental sampling captured a brown tide in Quantuck Bay, NY, United States from late May to early July in 2011 (Figure 1 and Table 1). As is common for this system, there were no significant positive correlations between physiochemical factors (nutrient concentrations, temperature, salinity, etc.) and cell densities over the course of the bloom (Table 2). The DIN:DIP ratio was consistently below Redfield during the sampling period (Figure 1). On June 14 (S1), cell densities reached 2.3 × 10^6^ cells mL^-1^, representing the peak of the bloom (Figure 1 and Table 1). Two days later, on June 16 (S2), the cells remained highly dense (1.9 × 10^6^ cells mL^-1^), but on June 22nd (S3), the cell densities rapidly decreased to about 7.0 × 10^5^ cells mL^-1^ (Figure 1 and Table 1). Therefore, S1 and S2 represent the peak of the bloom where densities were highest, while S3 represents the beginning of bloom termination, where cell densities were rapidly declining.

**Table 2:** Pearson correlation coefficients between *A. anophagefferens* cell densities versus assayed physical and chemical parameters.

	NO_3_	NH_4_	PO_4_	DIN^∗^	DON^∗^	DOP^∗^	DIN:DIP^∗^	DON:DOP	TN:TP^∗^
Pearson Coefficient	0.2834	–0.7444	0.5507	–0.1495	0.0657	–0.6949	–0.3255	0.6047	0.2422
*p*-value	0.496	0.034	0.157	0.724	0.877	0.056	0.432	0.112	0.563

*^∗^DIN, dissolved inorganic nitrogen; DIP, dissolved inorganic phosphorus; DON, dissolved organic nitrogen; DOP, dissolved organic phosphorus; TN, total nitrogen; TP, total phosphorus.*

Validation of the metatranscriptome data identified that the majority (>90%) of mapped reads from the three samples (S1, S2, S3) were specific to *A. anophagefferens* with the remaining reads mapping to potentially co-occurring species (Supplementary Table S1). Overall mapping rates were similar between *A. anophagefferens* strains and within 3% of each other (57% of total reads mapped to CCMP1850 while 59.9% mapped to CCMP1984 contigs). This overall mapping rate of around 60% is consistent with expectations from previous eukaryotic metatranscriptome studies across a range of environments (e.g., Alexander et al., 2015).

**FIGURE 2:**
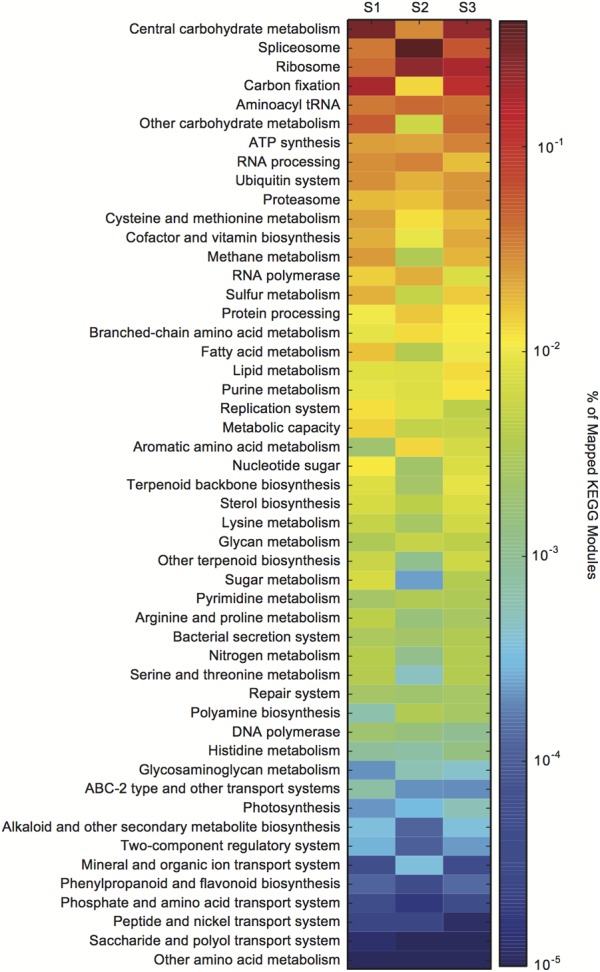
*Aureococcus anophagefferens* transcriptional changes across *in situ* samples (S1–S3). Colors indicate the relative expression of different KEGG functional classes (e.g., central carbohydrate metabolism) calculated by normalizing the library-normalized reads to the total number of KEGG annotated reads in a sample.

The metatranscriptome data were assigned KEGG orthology (KO) to visualize the expression of KO gene families within a KEGG module from bloom peak (S1 and S2) through the initial decline (S3) (Supplementary Dataset S1). There were dramatic transcriptional shifts between all samples (Figure 2). The splicesome, RNA processing, carbon fixation, and central carbohydrate metabolism categories all varied between S1 and S2, despite consistent cell densities (Figure 2). Compared to S1 and S2, there were changes in the relative expression of the lipid processing, purine metabolism, and proteasome categories in S3 (Figure 2).

**FIGURE 3:**
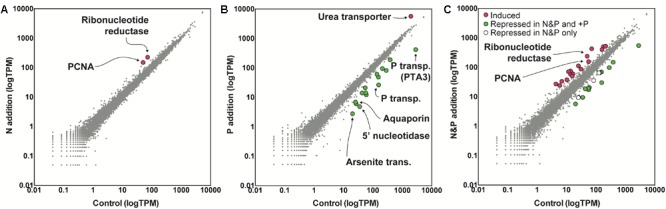
Gene expression values in tags per million (TPM) in the nitrogen (N) addition (left) **(A)**, phosphorus (P) addition (middle) **(B)**, and N&P addition (right) **(C)** experiments relative to a no nutrient added control. The nutrient amendment experiment was started during bloom decline at S3 as noted in Figure 1. Significance was determined with ASC (fold change > 2, post-*p* > 0.95). Red indicates genes with significantly increased expression and green indicates genes with significantly decreased expression in the nutrient addition treatments versus the control. Note the log scale of the axes.

To examine the role of nutrients in bloom dynamics, an *in situ* nutrient amendment experiment was performed on June 22nd that included four treatments: a no nutrient addition control, +NH_4_^+^ (+N), +PO_4_^-3^ (+P), and +NH_4_^+^+PO_4_^-3^ (+N&P). After 24 h, growth rates were negative for the control and the +N treatments (Figure 1). Only two genes were significantly increased in abundance in the +N treatment: a ribonucleotide reductase [protein ID (PID): 30730] and a DNA-binding/proliferating cell nuclear antigen (PID: 70163) – important in DNA synthesis and repair (Figure 3). No genes were significantly decreased in the +N treatment.

In contrast to the +N treatment, growth rates were positive in the +P and +N&P treatments (Figure 1). There were 15 genes with significantly decreased abundance in the +P treatment, many of which are involved in P scavenging/metabolism (Figure 3). Among these 15 genes, there were two phosphate transporters (PID: 10532 and 70513) and a 5′-nucleotidase (PID: 10515). Additionally, an arsenite translocating ATPase (PID: 64509) was also decreased upon phosphate addition (Figure 3). Only one gene was significantly increased in the +P treatment, a urea transporter (PID: 71789). A total of 16 genes had decreased abundance in the +N&P treatment. Of these, 13 were the same genes that had decreased abundance in the +P treatment (Figure 3). The urea transporter was not significantly increased in the +N&P treatment, which received +NH_4_^+^.

**FIGURE 4:**
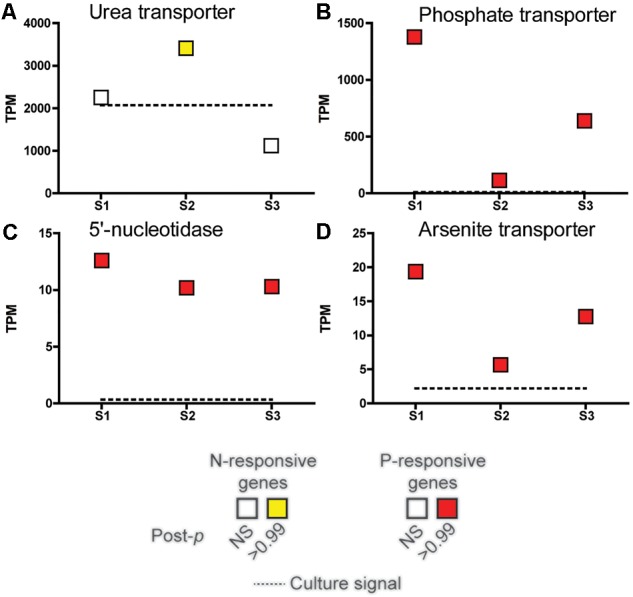
Expression patterns in tags per million (TPM) at peak bloom (S1 and S2) and bloom decline (S3) of select nutrient-responsive genes detected in the incubation experiments. The TPM of the urea transporter **(A)**, phosphate transporter (PTA3) **(B)**, 5′-nucleotidase **(C)**, and arsenite-translocating ATPase Arsenite transporter **(D)** highlighted in Figure 3 are plotted. Significance was determined with ASC (fold change > 2, post-*p* > 0.99). Red and yellow indicates that the transcript was significantly more abundant while green and blue indicate significantly less abundant (none shown here), with a fold change greater than or equal to 2 relative to a nutrient replete culture control of *A. anophagefferens* (dashed line). NS indicates the transcript was not significantly different.

A subset of genes that showed significant changes in abundance in the nutrient incubation experiment were examined across the field samples spanning the bloom peak (S1 and S2) and its demise (S3) (Figure 4). The urea transporter was detected in all field samples, with TPMs (tags or reads per million) in the thousands across all samples, making it one of the genes with highest relative abundance in this study (Figure 4). However, the urea transporter was only significantly elevated (ASC: fold change ≥ 2; post-*p* > 0.95) relative to a replete culture control at S2 (Figure 4). In contrast, the phosphate transporter, 5′-nucleotidase, and arsenite translocating ATPase were significantly increased in all field samples relative to the replete culture control (ASC: fold change ≥ 2; post-*p* > 0.99).

## Discussion

Brown tides caused by *A. anophagefferens* occur regularly in many coastal regions, particularly along the eastern coast of the US (Gobler and Sunda, 2012). Despite many years of study, predictive abilities and management of brown tides remains a challenge (Gobler and Sunda, 2012). The focus of this study was to examine potential drivers of brown tides, with the emphasis directed toward controls on peak cell densities and bloom termination. As is common for Quantuck Bay NY, there were no significant positive correlations between physiochemical factors and cell densities over the course of the bloom. While the DIN:DIP ratio was consistently below Redfield, suggesting N control, *A. anophagefferens* can take up and metabolize certain DON and DOP substrates (Berg et al., 2002; Mulholland et al., 2002; Gobler et al., 2004; Wurch et al., 2011a), and so variations in nutrient bioavailability could influence cellular physiology in a way that is not predictable from the DIN:DIP ratio. Here, metatranscriptome profiling was used to evaluate cellular responses as a complement to nutrient dynamics, with a focus on peak cell densities and bloom decline. Although transcriptome data do not always reflect phenotypic changes, transcriptional changes are strongly linked to resultant changes in proteins, activities, and thus phenotype in this species (Wurch et al., 2011a; Frischkorn et al., 2014). As a result, we refer to metatranscriptome data as an indicator of physiology herein.

### Broad-Scale Transcriptional Changes During Peak Bloom and Bloom Decline

Global transcriptional patterns of *A. anophagefferens* were variable during both peak bloom conditions (S1 and S2) and bloom decline (S3) (Figure 2). At peak bloom densities, *A. anophagefferens* was undergoing large rearrangements in expression. For example, in S2 there appeared to be relative expression increases in the splicesome and RNA processing categories, and relative expression decreases in carbon fixation and central carbohydrate metabolism when compared to S1, despite what appeared to be stable conditions in the physiochemical environment and cell densities. These changes suggest reduced carbon fixation and metabolism at similar cell density, although more work is required to definitively link these changes in transcription to changes in phenotype or physiology for these aspects of metabolism.

Compared to S1 and S2, there were increases in the relative expression of lipid processing, and purine metabolism in S3. It has recently been shown that *A. anophagefferens*, and other eukaryotic phytoplankton, modulate intracellular metabolites related to purine metabolism when P-deficient (Kujawinski et al., 2017). Eukaryotic phytoplankton are also well known to remodel their membrane lipids in response to P deficiency (Van Mooy et al., 2009). The transcriptional remodeling observed at S3 is consistent with a potential role for P in bloom demise, but more work in understanding how these various transcript signals are regulated is required to evaluate how these global transcriptional changes may be linked to the factors driving bloom decline. Regardless, the apparent metabolic priorities of *A. anophagefferens* were strikingly variable during both peak bloom conditions (S1 and S2) and bloom decline (S3).

Based on validation work, the global shifts in transcription are not driven by errant mapping to other species, as mapping was highly specific and reconstructed the majority of the *A. anophagefferens* gene models. Some strains of *A. anophagefferens* are acutely toxic to bivalves and copepods (e.g., strain CCMP1850), while others are not (e.g., strain CCMP1984) (Harke et al., 2011), however mapping of field data was strikingly consistent between these two strains, suggesting variation in transcriptional patterns is not driven by variation in strain abundance. This is consistent with culture studies that found similar transcriptional responses when both strains were exposed to low N and low P conditions (Frischkorn et al., 2014). Although this strain comparison does not exclude the presence of multiple strains in the field, these data suggest that strain heterogeneity would not drive the variability observed here. The transcriptional variation observed here may be associated with “bottom up” type controls like the speciation of organic matter, rapid and differential cycling rates of nutrients, or other factors that would not be resolved from the bulk parameters used here to characterize the water column. Such module-level transcriptional variation has previously been observed in field populations of diatoms (Alexander et al., 2015). Naturally, the broad-scale transcriptional changes observed here could also be influenced by “top down” controls, like viral infection and lysis. There was evidence of active transcription of the *A. anophagefferens* giant virus AaV in S1, S2, and S3 (Moniruzzaman et al., 2017), suggesting that some of the transcriptional changes observed here could reflect modulation of host metabolism with infection.

### Transcriptional Patterns and Growth at Bloom Decline Are Modulated by Phosphate

To examine the role of nutrients in bloom dynamics, an *in situ* nutrient amendment experiment was performed during bloom decline. In the ammonium addition treatment (+N), growth rates were negative, suggesting that N was not limiting. Although high levels of ammonium can be toxic to *A. anophagefferens*, it can readily support growth at the concentrations used here (Taylor et al., 2006; Berg et al., 2008; Wurch et al., 2014). In culture, *A. anophagefferens* exhibits a broad transcriptional response to N deficiency, which is tightly modulated by N supply and form (Berg et al., 2008; Wurch et al., 2011b). Yet, here the addition of ammonium had little effect on *A. anophagefferens* gene expression patterns with only two genes showing significant increases, and no genes showing significant decreases, in expression. Thus, the metatranscriptome data reinforce the growth data concluding that N deficiency was not a factor controlling bloom decline.

In contrast to the +N treatment, growth rates were positive in the +P and +N&P treatments, suggesting P supply was controlling the growth rate of *A. anophagefferens* during this experiment. There were 15 genes that were significantly repressed upon P addition. Many of these genes were involved in P scavenging/metabolism, including two phosphate transporters and a 5′-nucleotidase. One of these phosphate transporters, PID: 10532, and the 5′-nucleotidase (PID: 10515) are highly responsive to P supply in culture, with increased abundance observed in *A. anophagefferens* cultures under acute P deficiency at both the RNA and protein level (Wurch et al., 2011a,b). Additionally, an arsenite translocating ATPase (PID: 64509), shown to be regulated by P in culture (Frischkorn et al., 2014), was also decreased upon phosphate addition. Arsenate can act as a phosphate analog, with inadvertent incorporation of arsenate leading to toxicity (see Tawfik and Viola, 2011 for review). *A. anophagefferens* blooms in anthropogenically-influenced estuaries and thus may be vulnerable to arsenic toxicity, particularly when P concentrations are low. The expression patterns of this arsenite translocating ATPase mirror those of the P-regulated phosphate transporter mentioned above. The synchronous decrease of a full suite of known P-regulated genes suggests that natural populations of *A. anophagefferens* were P-deficient during the post-peak bloom period. The addition of phosphate alleviated that P deficiency, leading to an increased growth rate and repression of genes considered markers for P deficiency. Collectively, these data support the role of P in controlling bloom dynamics.

Only one gene, a urea transporter, was significantly increased upon P addition in the +P treatment. A previous culture experiment with *A. anophagefferens* demonstrated that expression levels of this urea transporter increase during both acute N deficiency and if the population is shifted to growth on urea (Berg et al., 2008). It may be that N is secondarily limiting in this system such that P addition at S3 pushes the community to N deficiency (see review by Saito et al., 2008), or to a switch to the use of DON sources like urea. Previous research has emphasized the importance of urea as an N source for *A. anophagefferens* bloom populations (Mulholland et al., 2002; Kana et al., 2004), and this is supported by the dynamics of the urea transporter observed herein.

A total of 16 genes had decreased abundance in the +N&P treatment. Of these, 13 were the same genes that had decreased abundance in the +P treatment. However, the urea transporter did not significantly increase in abundance as it had in the +P treatment, consistent with N being secondarily limiting, and the urea transporter potentially being an indicator of N deficiency. The higher growth rates in the +N&P treatment were consistent with increased expression of several genes involved in DNA synthesis and replication that were not significantly increased in the +P treatment alone.

A subset of genes with corroborating culture data (Berg et al., 2008; Wurch et al., 2011b; Frischkorn et al., 2014) and that showed significant changes in abundance in the nutrient incubation experiment were examined across the field samples spanning the bloom peak (S1 and S2) and its demise (S3). The urea transporter was detected in all field samples at high relative abundance. Although caution must be employed in the extrapolation of culture signals to the field, the urea transporter signal was only significantly higher than replete culture controls growing on nitrate at S2. This S2 signal corresponds with both the lowest DIN:DIP ratio of the bloom as well as the lowest total DON. Previous work has shown that this gene is induced under acute N deficiency, but N type also has a strong effect on expression (Berg et al., 2008). In culture, the urea transporter was 20-fold more abundant when *A. anophagefferens* was supplied with urea as its sole N source compared to nitrate or ammonium (Berg et al., 2008). Therefore, it is difficult to resolve whether the signals and patterns here are indicative of N deficiency, or a switch to growth on urea. However, a lack of response from other N-related genes that are known to be induced under N deficiency, such as various N transporters (Berg et al., 2008) and a xanthine/uracil/vitamin C permease (Wurch et al., 2014), at least suggests this brown tide was utilizing urea as its nitrogen source rather than experiencing N deficiency, particularly at S2. In order to fully resolve this, the urea transporter would need to be functionally characterized and its regulation patterns in several strains constrained through detailed culture experiments on different nitrogen sources. Regardless, the results here suggest *A. anophagefferens* takes advantage of urea as an N source when inorganic forms are exhausted. These findings are consistent with urea enrichments that have been shown to stimulate brown tides *in situ* (Kana et al., 2004).

DIN:DIP ratios over the course of this study were well below the Redfield ratio. Solely relying upon the observed DIN:DIP ratio might have led to the prediction that *A. anophagefferens* was N-deficient during this study, yet gene expression and growth response suggest otherwise. All of the P-regulated genes were significantly higher than the signal typically detected in nutrient replete, exponentially growing cultures of *A. anophagefferens* CCMP 1984, with similar patterns in the signal across the sample set (particularly for the transporters). These consistent signals of P deficiency across the sample set suggest that P is a controlling factor during both the peak bloom period and the bloom termination in S3. These data highlight the value of tracking transcriptional markers of nutritional physiology, as the geochemistry would not have predicted P as a driving factor in the bloom dynamics. Strikingly, the phosphate transporter and arsenite translocating ATPase had signals orthogonal to that of the urea transporter, suggestive of a dynamic interplay between N and P in this system. Again, these dynamics would not be resolved from the geochemistry alone. In sum, the modulation of the P-regulated genes in *A. anophagefferens* across the bloom phases, and the S3 nutrient incubation study, collectively support the role of P as a controlling factor in the dynamics of this harmful bloom. Although P is known to be a controlling factor in the bloom dynamics of other HABs, these are most frequently observed in freshwater (O’Neil et al., 2012). For example, concentrations of bioavailable P were a controlling factor over a 2011 record-breaking bloom of the harmful cyanobacteria *Microcystis* in Lake Erie (Michalak et al., 2013; Harke et al., 2016). A 2009 brown-tide in Quantuck Bay showed evidence of P deficiency signals during bloom initiation, with targeted expression studies of PTA3 (PID: 10532) (Wurch et al., 2014) suggesting that the role of P deficiency in this system may be critical in controlling bloom initiation, capping peak cell densities, and controlling bloom decline, depending on the year and local conditions.

The expansion of human populations along coastlines has led to a progressive enrichment in turbidity, organic matter, including organic nitrogen, and metals in estuaries (Gobler et al., 2011). The expressed gene complement of *A. anophagefferens* at peak cell densities was consistent with the hypothesis proposed by Gobler et al. (2011) that this species occupies a niche characterized by conditions (low light, high organic matter, etc.) that have become increasingly prevalent in anthropogenically-influenced estuaries. In systems which host *A. anophagefferens* blooms, anthropogenic nutrient loading promotes algal growth and, as a result, elevated levels of organic matter and turbidity, providing a feedback loop that could further promote favorable bloom conditions (Gobler et al., 2011). Traditionally, strategies to mitigate estuarine algal blooms have targeted reductions in N only, as this element has been shown to control algal biomass in marine ecosystems (Howarth and Marino, 2006). In regions of NY where *A. anophagefferens* blooms occur, however, a substantial increase in the volumes of wastewater discharged to groundwater has enriched the concentrations of N discharged from groundwater into estuaries by more than 200% since 1980, while P levels have largely remained unchanged (Suffolk County Comprehensive Water Resources Management Plan, 2015). The asymmetric delivery of nutrients is likely a factor that has altered this once strongly N-limited system (Ryther and Dunstan, 1971) into one that today can drive dense blooms of *A. anophagefferens* into P deficiency. Further, this suggests a dual nutrient mitigation strategy that restricts the delivery of both N and P into estuaries may be most effective for mitigating blooms of this HAB (Conley et al., 2009). Regardless, given the known interactions between bloom dynamics and N geochemistry in this system, the apparent role of P in limiting growth rate and in bloom decline was unexpected and would not have been resolved without directly assaying *A. anophagefferens* physiology with the combination of nutrient amendment experiments and metatranscriptomics used herein. As we move forward, continued anthropogenic modification may further the unbalanced delivery of N and P into coastal ecosystems, and applying this metatranscriptomics approach to additional brown tide events, and other HABs, may help determine if similar dual nutrient management strategies should be more widely considered.

## Data Availability Statement

All project sequence reads are available at the National Center for Biotechnology Information (NCBI) under accession number SRP072764 and bioproject number PRJNA315054.

## Author Contributions

LW, CG, and SD designed the study. CG collected the field samples and data, and performed incubation experiments. LW and SH prepped samples for sequencing and performed culturing work. HA and KF performed bioinformatics analysis. LW, CG, and SD analyzed the data and wrote the manuscript.

## Conflict of Interest Statement

The authors declare that the research was conducted in the absence of any commercial or financial relationships that could be construed as a potential conflict of interest.

## Footnotes

1 http://www.ncbi.nlm.nih.gov/genome/genomes/437

2 http://genome.jgi.doe.gov/Auran1/Auran1.home.html
